# Risk of Myocardial Infarction in HIV Patients: A Systematic Review

**DOI:** 10.7759/cureus.31825

**Published:** 2022-11-23

**Authors:** Maram M Alsheikh, Ahmed M Alsheikh

**Affiliations:** 1 Department of Cardiology, University Hospital Southampton NHS Foundation Trust, Southampton, GBR; 2 Faculty of Medicine, Suez Canal University, Ismalia, EGY; 3 Department of Internal Medicine, Dr. Abdul Rahman Al Mishari Hospital, Riyadh, SAU; 4 College of Medicine, Almaarefa University, Riyadh, SAU

**Keywords:** myocardial infarction, acute myocardial infarction, incidence, risk, hiv

## Abstract

Human immunodeficiency virus (HIV) is a retrovirus that is associated with mortality in the final stage. The advancement of antiretroviral therapy (ART) improved the life expectancy of patients with HIV. However, the long age of such patients is associated with different comorbidities such as cardiovascular diseases. Also, HIV therapy increased the concern about cardiovascular diseases. This systematic review aims to assess the risk of myocardial infarction (MI) among patients with HIV by reviewing the previous studies conducted on this subject. Research gate, Google Scholar, and PubMed databases were explored starting from 2012 till 2022. The keywords used for the searching process included "HIV, MI, AMI, Association, Correlation, and Risk." The inclusion criteria were original articles conducted on HIV patients and reported MI, written in English language, and available in full text. A total of 1,570 articles were obtained, but only seven articles met the inclusion criteria. The included studies were published between 2012 and 2019 and involved a total number of 496,600 participants; there were 266,274 who had HIV infection, with a sample size ranging from 1,147 to 252,150. The incidence of MI is higher among HIV compared to the general population. The risk factors associated with MI among HIV patients, as found in our analysis, included male gender, viral load of HIV, low CD4 count, higher CD8 count, and types of ART.

## Introduction and background

The prevalence of the human immunodeficiency virus (HIV) is increasing [1]. HIV is a retrovirus identified as two strains: HIV-1 was commonly spread from patients with acquired immunodeficiency disease syndrome (AIDS), and the second strain is HIV-2, and it was found primarily in West Africa [2]. HIV infection is characterized by four phases: acute or primary infection phase, asymptomatic or clinical latency phase, initial or early symptomatic phase, and AIDS. The natural progression of HIV disease begins with the transmission of an individual and ends with the death of that individual without antiretroviral therapy (ART) [2].

The advancement of ART, diagnosis, management, and prevention of deadly infections in HIV led to an increase in the life expectancy of patients. As a result, HIV patients are less prone to dying from infectious disease; however, they become more prone to experiencing cardiovascular and metabolic diseases [3]. The burden of cardiovascular diseases among persons with HIV is increasing [1]. Cardiovascular disease risk increased by 40-75% among individuals infected with HIV compared to non-infected individuals [4].

Cardiovascular disease is a global health problem with high morbidity and mortality rates. Myocardial infarction (MI) is the necrosis in the myocardium due to the lack of oxygen supply in the heart, which cannot be supplied by the coronary arteries [5]. It was found that patients infected with HIV are more predisposed to suffer from acute myocardial infarction (AMI), and this became even more pronounced among patients who were receiving ART [3]. Therefore, this systematic review was performed to assess the risk of MI among patients with HIV.

## Review

This analysis follows the PRISMA (Preferred Reporting Items for Systematic Reviews and Meta-Analyses) checklist guidance for systematic review and meta-analysis [6]. Electronic databases were revised to select eligible research articles between the year 2012 and the year 2022, including Research gate, PubMed, and Google scholar databases.

Search strategy

Various terms and keywords were used for searching purposes, including "HIV, MI, AMI, Association, Correlation, and Risk." All the titles and abstracts produced from this primary search were revised thoroughly to avoid missing any potential study. The obtained articles were then examined to choose only original research studies reporting MI or AMI among HIV patients. All articles from all countries were eligible, but only articles in English were included, and then these studies were included in the second stage.

Eligibility criteria

**Figure 1:**
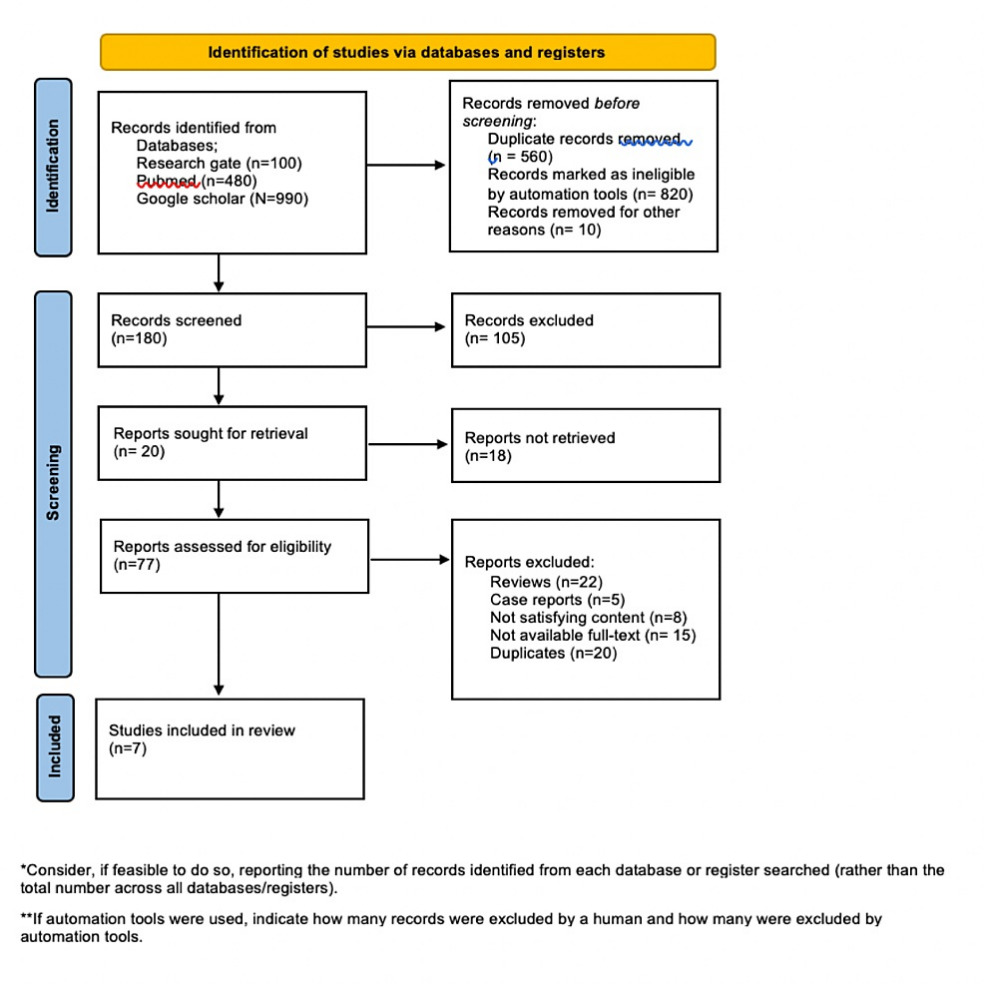
The flowchart of eligibility criteria

The second step involved evaluating the abstracts manually to select the relevant studies for revision. The inclusion criteria were studies that reported MI among HIV patients, and that studies compared MI among HIV patients and the general population. The final stage involved gathering the pre-defined data from the final record of the eligible studies and summarizing them. Reviews, case reports, and studies containing incomplete or overlapped data were excluded. Also, unavailable full-text articles were excluded. The full description of the search strategy is shown in Figure 1.

Data review

An Excel sheet was specially designed by two reviewers to be used for data extraction. Chosen data from eligible articles were transferred to the Excel sheet and revised. Any research article published by one research group examining similar variables was reviewed for any potential duplication.

Results

There were seven articles that met the eligibility criteria and were included in our analysis [7-13] (Table 1). According to the publication year, the studies were published between 2019 and 2012. The study design was not reported in two studies [7,9], whereas the remaining studies reported different designs and included longitudinal [8], observational [10], cohort [11], prospective longitudinal cohort [12], and nested case-control [13]. The sample size ranged between 1,147 [13] and 252,150 [11], with a total of 496,600 subjects. Of the total number, there were 257 MI cases, 230,069 HIV-negative individuals, and the remaining frequency (266,274) represented HIV patients. Five studies included one group of patients, which involved HIV patients [7-9,10,12], whereas two studies included two groups (one study included HIV patients and HIV-negative individuals [11], and the other study included case group of MI patients and control group of HIV patients [13]).

**Table 1:** Summary of the included studies HIV; human immunodeficiency virus, cART; combination antiretroviral therapy, SIR; standardized incidence rate, MI; myocardial infarction, AMI; acute myocardial infarction, PLWH; people living with HIV, ARIC; atherosclerosis risk in communities, aIRR; adjusted incidence rate ratio, CVD; cardiovascular disease, ABC; abacavir, CvRFs; cardiovascular risk factors; HR, hazard ratio; RR, relative risk

Author	Years	Study design	Sample size	Population	Objective	Results
Balde et al. [7]	2019	-------	71,204 HIV patients	HIV patients	Identify trends in the incidence of MI among individuals living with HIV	HIV infection in 2000-2002 (early cART era) was found in 43,628 individuals; 174 (0.33%) had MI. HIV infection in 2003-2005 (intermediate cART era) was found in 51,007; 221(0.43%) had MI. HIV infection in 2006-2009 (late cART era) was found in 58,866; 295 (0.5%) had MI. The incidence of MI among males was 1.16%, and among females it was 0.33%. Regarding MI patients, males were more predominant compared to females during the three periods. The trends were different for males and females with a decreasing trend in SIR in men and no change in women. The standardized incidence rates of MI changed remarkably across the three periods among HIV males, HIV females, and among men and women of the general population, remaining higher in the HIV-infected population compared to the general population. In both genders, the risk was no longer elevated among persons with CD4 ≥ 500/μL and controlled viral load on cART. Age at the diagnosis of MI was remarkably younger compared to the general population, especially among women (-6.2 years, p<0.001; men: -2.1 years, p = 0.02). In HIV-1 patients, absolute rate difference and RRs and trends of MI were different between males and females and there was no additional risk among individuals on effective cART.
Masia et al. [8]	2018	Longitudinal	10,760 PLWH	PLWH	To analyze time trends in the standardized incidence rate (SIR) of AMI in PLWH in Spain from 2004 to 2015, and compared them with trends in the general population.	The SIR of AMI in 2004–2015 was 237.92 and 66.75 per 100 000 patient-years in male and female PLWH, respectively. There was a reduction in the sIR of AMI in male PLWH from 279.02 per 100 000 person-years in 2004–2009 to 222.13 per 100 000 person-years in 2010–2015. Compared with the general population, the SIR ratio was 1.41 in 2004–2009 and 1.28 in 2010–2014. AMI occurrence was associated with older age (p < 0.066 for each 10-year age stratum ≥ 35 years compared with the 25- to 34-year stratum), lower CD4 count (p < 0.04 for CD4 strata > 350 cells/lL compared with the 0-100 cells/lL stratum), higher plasma HIV RNA (p < 0.001), and the period 2004–2009 (p < 0.001). There has been a decreasing incidence of AMI in PLWH associated with improvements in viriological and immune status, but the incidence of AMI remained higher compared to the general population.
Drozd et al. [9]	2017	---------	29,169 HIV patients	HIV-infected individuals	To assess the incidence of T1MIs and risk related to HIV-specific and traditional factors among the individuals in the North American	The incidence rate for type 1 MI was 2.57 (2.3-2.86) per 1,000 person-years. The aIRR was considerably higher compared with individuals in ARIC (1.30; (1.09-1.56). In multivariable analysis among HIV-infected persons and including traditional Cardiovascular diseases risk factors, the rate of type 1 myocardial infarction increased with decreasing CD4 count [≥500 cells/mL: aIRR = 1.32 (0.98 to 1.77); 200–349 cells/mL: aIRR = 1.37 (1.01 to 1.86); 100–199 cells/mL: aIRR = 1.60; 100 cells/mL: aIRR = 2.19.]In multivariable analysis among HIV-infected persons and including traditional Cardiovascular diseases risk factors, the rate of type 1 myocardial infarction increased with decreasing CD4 count [≥500 cells/mL: aIRR = 1.32 (0.98 to 1.77); 200–349 cells/mL: aIRR = 1.37 (1.01 to 1.86); 100–199 cells/mL: aIRR = 1.60; 100 cells/mL: aIRR = 2.19]. Risk associated with detectable HIV RNA [400 copies/mL: aIRR = 1.36 was remarkably increased only when CD4 was excluded. There was a higher incidence of T1MI in HIV-infected persons and the increased risk was associated with detectable HIV RNA and lower CD4 count; therefore, early initiation of antiretroviral treatment and management of traditional CVD risk factors are necessary to maximally decrease MI risk.
Sabin et al. [10]	2016	Observational	497,17 patients	HIV-1-positive patients from 11 cohorts	To characterize changes to the use of abacavir and examine changes to the association between abacavir and MI	Use of ABC increased from 10% to 20% in 2000 to 2008. By February 1, 2013, there were 941 MI events occurred in 367,559 person-years. Current abacavir use was correlated with a 98% increase in MI rate with (RR = 1.98) with no difference in the pre- (1.97) or post- (1.97) March 2008 periods (interaction p = 0.74). There is an association between ABC use and the risk of MI, although there is a reduction in the channelling of ABC for patients at higher CVD risk since 2008.
Silverberg et al. [11]	2013	Cohort	22,081 HIV-positive patients. 230,069 HIV-negative individuals	HIV-positive patients and HIV-negative individuals	To identify the association of HIV infection and immunodeficiency with the risk of MI	The crude MI incidence rate per 100,000 person/years was 283 and 165 for HIV-positive and HIV-negative persons, respectively, with an adjusted RR = 1.4. Compared with HIV-negative patients, MI rates were similar for HIV-positive patients with recent CD4 ≥ 500 cells/microliter (RR = 1.18) and those with nadir CD4 ≥ 500 cells/microliter (RR = 0.85). Among HIV-positive patients, nadir CD4 was the only HIV-specific factor associated with MI (RR per 100 cells = 0.88), whereas duration of protease inhibitors, prior ART use, recent CD4 and HIV RNA, and non-nucleoside reverse transcriptase inhibitors were not associated with MIs. HIV-positive individuals with recent or nadir CD4 ≥ 500 cells/ micorliter had similar MI rates compared with HIV-negative subjects. Lower nadir CD4 is independently associated with MI; therefore, ART early initiation is recommended.
Freiberg et al. [12]	2013	Prospective longitudinal cohort	82,459 patients	HIV-positive veterans aging individuals	To investigate the association between HIV and the increased risk of AMI after the adjustment for all standard Framingham risk factors	During 5.9 years, a total of 871 AMI events recorded. The mean AMI events per 1,000 person-years across three decades was considerably higher for HIV patients in comparison with uninfected veterans: for those aged 40 to 49 years, 2.0 vs 1.5; for those aged 50 to 59 years, 3.9 vs 2.2; and for those aged 60 to 69 years, 5.0 vs 3.3 (p ˂ 0.05 for all). After adjustment, HIV-positive veterans showed an elevated risk of incident AMI compared with uninfected veterans (HR = 1.48). An excess risk was among those achieving an HIV-1 RNA level ˂500 copies/mL compared with uninfected veterans in time-updated analyses (HR, 1.39). Infection with HIV is associated with a 50% elevated risk of AMI beyond that explained by recognized risk factors.
Lang et al. [13]	2012	Nested case-control	257 case patients (MI), 884 controls (HIV)	Case group involved MI patients and control group included HIV-infected patients	To investigate the role of HIV virological and immunological parameters, in addition to CvRFs and ART exposure, on the risk of MI in HIV-infected individuals	A low CD4 T-cell nadir, plasma HIV-1 RNA level >50 copies/mL, and a high CD8 T-cell count were independently correlated with an elevated risk of MI, with respective odds ratios of 1.51 (95% confidence interval: 1.09–2.10), 0.90 (.83–.97) per log2 unit, respectively. Independent of cardiovascular risk factors and antiretroviral therapy, HIV replication, a low CD4 T-cell nadir, and a high current CD8 T-cell count are associated with an elevated risk of MI in infected persons.

The objectives of the studies involved the identification of the trends and incidence of MI among HIV patients [7] and comparison to the general population [8], assessment of incidence and risk of MI among HIV patients [9], and changes to the association between abacavir and MI [10]. The objectives also included identification of the association between HIV and immunodeficiency on MI risk [11], the association between HIV and increased risk of AMI [12], and the role of HIV parameters and cardiovascular risk factors and ART exposure on the risk of MI [13].

Regarding results, the incidence of MI was determined in three durations (2000-2002, 2003-2005, and 2006-2009); the incidence was 0.33%, 0.43%, and 0.5%, reflecting an increase in the incidence with time [7]. Another study estimated the standardized incidence rate ratio during two periods; it was 1.41 in the first period (2004-2009) and reduced to 1.28 in the second period (2010-2014), but still higher than among the general population [8]. One study reported that the incidence rate of type 1 MI (T1MI) was 2.57/1,000 persons, with an adjusted incidence rate ratio higher among HIV patients compared to the general population [9]. Another study estimated the crude incidence among HIV patients compared to negative controls; the incidence among patients was 2.83/1,000 person-years, whereas that of negative controls was 1.65/1,000 person-years with a relative risk (RR) of 1.4 [11]. One study reported the number of events of MI, and there were 941 MI events among 367,559 person-years [10].

The incidence and risk of MI regarding gender were reported in two studies [7,8]. One study showed that the incidence of MI among males was higher compared to females; 1.16%. versus 0.33%, respectively, and males were predominant compared to females during the three periods of studies. Additionally, there was a decreasing trend in standardized incidence rate among males with no change among females [7]. The other study reported an incidence among males higher compared to females; the incidence among males was 2.37/1,000 person-years, and among females it was 0.66/1,000 person-years. There was a decrease in the standardized incidence rate among males during two periods; the incidence reduced from 2.79/1,000 person-years in the first period (2004-2009) to 2.22/1,000 person-years in the second period (2010-2015) [8].

Regarding the risk factors of MI, one study revealed that MI was significantly diagnosed among younger age patients, especially among women, whereas the risk was not elevated among individuals with those with the controlled viral load on combination antiretroviral therapy (cART) and those with CD4 ≥ 500/μL. Effective cART protected patients from additional risk [7]. One study was conducted on aging veteran participants. The events of AMI were significantly higher among HIV-infected patients compared to uninfected participants, with an incidence increasing with age; the incidence was 2 versus 1.5 for those aged 40-49 years, and it increased to 5 versus 3.3 for those aged 60-69 years. Additionally, the excess risk remained associated with HIV RNA levels [12]. On the other hand, another study showed that AMI was significantly associated with older age (P˂0.06), higher plasma HIV RNA (p˂0.001), lower CD4 count (p˂0.04), and the period 2004-2009 (p˂0.001). The risk and incidence of AMI were reduced by improving immune and virological status [8]. The rate of T1MI increased with decreasing in CD4 count; the reduction of CD4 count resulted in an increase in the adjusted incidence rate ratio. The risk of T1MI was associated with detectable HIV RNA [9]. The increased risk of MI was associated with plasma HIV RNA level (HIV replication), low CD4 nadir, and high CD8 count [13]. MI rate was associated with low nadir CD4 with an RR of 0.88 for 100 cells. On the other hand, prior ART use, duration of protease inhibitors, non-nucleoside reverse transcriptase inhibitors, HIV RNA, and recent CD4 were not associated with MI risk [11]. One study assessed the impact of abacavir use and revealed that the current use of abacavir was associated with a 98% increase in MI rate [10].

Discussion

The pathogenesis of heart disease during infection of HIV is multifactorial with the interplay of host cardiovascular risk factors, HIV infection, and ART [14]. In this systematic analysis, we aimed to assess the risk of MI among patients with HIV and risk factors.

In our analysis, the incidence of MI in one study increased over time among HIV patients [7], whereas another study reported a reduction in incidence [8]. However, this may be attributed to the variation between the two studies in design, characteristics of patients, and medications used. Also, there was a variation regarding the incidence used for determination, such as standardized incidence rate, adjusted incidence rate ratio, and crude incidence. Thus, we can conclude that MI events occur more among individuals infected with HIV compared to non-infected ones. In a retrospective study, it was revealed that silent MI was increased among HIV-infected patients compared to the general population (11% vs. 5%, respectively) [15]. These findings were in agreement with our analysis.

In a study including 20 HIV patients admitted with acute coronary syndrome, it was found that the majority were suffering from MI, and 19 patients were males [16]. These findings indicate the predominance of males compared to females. In our analysis, only two studies reported the impact of age [7,8]; the incidence of MI among males was higher compared to females, but the reducing trend among males was obvious.

The impact of age was not previously reported; however, we found conflicts between the included studies regarding age and MI among HIV patients. One study showed that MI was significantly diagnosed among younger age patients [7]. Another study showed increased incidence with increasing age [12], and the last study showed that the association was not significant (p ˂ 0.06) [8]. However, this confection can be attributed to the variation of study design, characteristics of patients, their viral load, type of medication, and stage or type of MI, as some studies reported MI and others reported AMI.

A study from Brazil conducted on HIV patients showed that virologic suppression and preservation of CD4 count were important traditional risk factors for cardiovascular disease burden and determining incident cardiovascular disease event risk [17]. Another study revealed that cART use and high CD4 count were considerably associated with a reduced hazard of CDV [18].

In our analysis, CD4 count and RNA viral load were determinant factors for the risk of MI. Low CD4 count and nadir CD4 were associated with an increased risk of MI among HIV patients. Also, increased CD8, which reflects a reduction in CD4, is also associated with MI risk among HIV patients. The viral load of HIV can be controlled by ART. Patients with controlled viral load due to cART were at lower risk of MI. Therefore, the viral load of HIV is a determinant factor for MI risk, and it can be controlled through medication. However, the type of therapy is another determinant of MI risk. One study reported that controlled viral load with cART was associated with a lower risk of MI [7]. Also, another study reported that the use of ART, duration of protease inhibitors, and HIV RNA were not associated with MI risk [11]. However, one study revealed that abacavir use was associated with a 98% increase in MI rate [10].

cART has a dramatic impact on reducing mortality and morbidity associated with HIV-1 [19]. However, the impact of such medication raised concern regarding the risk of coronary heart disease [19], as we found in our analysis that abacavir increased the rate of MI [10]. Some cohorts suggested a positive correlation between ART, including specific drugs such as abacavir or drug classes such as protease inhibitors, and MI risk [20-22]. These reports were in agreement with our findings regarding abacavir; however, there are studies that do not prove the increase in cardiovascular risk of abacavir. Also, in our analysis, current guidelines recommend early initiation of ART after diagnosis [9,11], and emphasize the overall benefits of ART on cardiovascular risk in a previous study [17]. Therefore, the risk of MI may be dependent on specific medications for HIV.

The risk factors associated with MI among HIV patients, as found in our analysis, included a viral load of HIV, low CD4 count, higher CD8 count, and some types of ART.

In one study, it was found that the incidence of MI increased from 1.53/1,000 person-years among those not exposed to protease inhibitors to 6.01/1,000 person-years among those exposed to protease inhibitors. The study revealed an increased risk of MI in case of increased exposure to protease inhibitors, whereas there was no evidence of association for non-nucleoside reverse transcriptase inhibitors [19]. The previous was in contrast to our findings, where one study reported that the duration of protease inhibitors and non-nucleoside reverse transcriptase inhibitors were not associated with MI risk.

A systematic review containing 32 studies indicates that the pooled RR suggested that the patients with HIV have a greater risk of MI compared with uninfected subjects at RR of 1.73. Also, it was found that low CD4 count, high pVL, and certain ART characteristics such as cumulative ART exposure, including certain exposure to a specific ART regimen or class, were associated with a greater risk of MI [23]. Another systematic review and meta-analysis were conducted to identify the RR of cardiovascular disease among HIV patients. The analysis reported that not all ARTs were associated with increased risk; specifically, ritonavir, lopinavir, and abacavir were associated with the greater risk, and the RR of MI for protease inhibitor-based versus non-protease inhibitor-based ART was 1.41 [24].

Limitations, strengths, and recommendations

The limitations in this analysis, including the studies included, were not similar in design, and there were many incidence estimates (crude, adjusted, and standardized). The strength of the study includes highlighting the role of age in MI risk, and we reported several points, such as the incidence of MI among HIV patients and the risks for MI among such patients. Further studies are recommended to be conducted to assess the impact of age on IM risk among HIV patients, determine the impact of HIV therapy on MI risk, and determine the medications associated with higher risk.

## Conclusions

MI is associated with HIV infection, and its incidence is increasing, and it is more incident among HIV compared to the general population. Therefore, HIV can be considered a risk factor for MI. The risk factors associated with MI among HIV patients, as found in our analysis, included male gender, viral load of HIV, low CD4 count, higher CD8 count, and types of ART. Therefore, these factors can be used as predictors for MI among patients with HIV; this can help in prophylaxis against MI. On the other hand, there was a conflict regarding the age that requires further investigations.
